# A novel score for early prediction of urinary tract infection risk in patients with acute ischemic stroke: a nomogram-based retrospective cohort study

**DOI:** 10.1038/s41598-024-61623-0

**Published:** 2024-05-10

**Authors:** Qinqin Zhao, Pinpin Feng, Jun Zhu, Yunling Wang, Xiaojuan Zhou, Zhongni Xia, Danqing Wang, Yueyue He, Pei Wang, Xiang Li

**Affiliations:** 1 Department of Pharmacy, Tongde Hospital of Zhejiang Province, No. 234 Gucui Road, Xihu District, Hangzhou City, 310012 Zhejiang Province China; 2 Department of Neurology, Tongde Hospital of Zhejiang Province, Hangzhou, 310012 China; 3 School of Pharmaceutical Sciences, Hangzhou Medical College, Hangzhou, 311399 China; 4 School of Basic Medical Sciences & Forensic Medicine, Hangzhou Medical College, No. 8 Yikang Street, Lin’an District, Hangzhou City, 311399 Zhejiang Province China

**Keywords:** Urinary tract infections, Stroke, Prediction, Nomogram, Model, Urinary tract infection, Stroke

## Abstract

This study aimed to construct and externally validate a user-friendly nomogram-based scoring model for predicting the risk of urinary tract infections (UTIs) in patients with acute ischemic stroke (AIS). A retrospective real-world cohort study was conducted on 1748 consecutive hospitalized patients with AIS. Out of these patients, a total of 1132 participants were ultimately included in the final analysis, with 817 used for model construction and 315 utilized for external validation. Multivariate regression analysis was applied to develop the model. The discriminative capacity, calibration ability, and clinical effectiveness of the model were evaluated. The overall incidence of UTIs was 8.13% (92/1132), with *Escherichia coli* being the most prevalent causative pathogen in patients with AIS. After multivariable analysis, advanced age, female gender, National Institute of Health Stroke Scale (NIHSS) score ≥ 5, and use of urinary catheters were identified as independent risk factors for UTIs. A nomogram-based SUNA model was constructed using these four factors (Area under the receiver operating characteristic curve (AUC) = 0.810), which showed good discrimination (AUC = 0.788), calibration, and clinical utility in the external validation cohort. Based on four simple and readily available factors, we derived and externally validated a novel and user-friendly nomogram-based scoring model (SUNA score) to predict the risk of UTIs in patients with AIS. The model has a good predictive value and provides valuable information for timely intervention in patients with AIS to reduce the occurrence of UTIs.

## Introduction

Stroke is the 2nd leading cause of death, responsible for approximately 11% of total deaths globally^1^. Owing to its high morbidity, mortality, disability, and recurrence, stroke remains a tremendous public heath burden worldwide^2,3^. Ischemic stroke (IS) is the predominant subtype of stroke, accounting for 87% of stroke cases^4^. Medical complications are very common after an acute ischemic stroke (AIS)^5,6^. This is probably due to immunosuppression and the transfer of specific intestinal flora, which make patients with stroke more susceptible to infection^7^. Urinary tract infections (UTIs) are among the most significant medical complications in patients with stroke^5^. The occurrence rate of UTIs after stroke ranges from 2 to 27%, as reported^8–10^. Although UTI is a common cause of morbidity in the general medical population, patients with stroke have a higher risk of developing UTIs compared to them, leading to more significant consequences^11^ such as poor functional outcome, mortality, disability, extended hospital stay and increased medical care costs^12–14^. Therefore, developing simple and practical tools that can predict UTIs early after AIS is essential to enable timely medical intervention and precise therapeutic decisions.

Various methods have been attempted to predict post-stroke UTIs. Most previous studies have explored and demonstrated various independent high-risk predictors, including the National Institute of Health Stroke Scale (NIHSS) score, indwelling catheters use, female sex, diabetes mellitus, advanced age, among others^15–17^. Meanwhile, predictive models have been established for patients with high incidence rates of UTIs, such as pediatric urological surgery patients, young children with fever, neurointensive care patients, and patients under emergency care^18–21^. However, these models are not applicable to patients with stroke. To date, only a limited number of predictive models have been developed for stroke patients to predict UTIs. A logistic regression model was developed based on data from 186 AIS patients^22^, while another machine learning method model was developed using data from 110 patients with intracerebral hemorrhage^23^. Nevertheless, the relatively small sample sizes in both studies may limit their generalizability. Moreover, there is also a prediction model based on a machine learning method that includes a substantial sample size has been constructed; however, it was derived from immobile patients with stroke, encompassing both hemorrhagic and ischemic stroke^24^. Moreover, it should be noted that these models do not function as scoring systems, rendering them less user-friendly. Therefore, improved models for predicting UTIs in all AIS patients remain inadequate and are urgently needed.

In this study, we conducted an observational real-world cohort study to derive and externally validate a novel nomogram-based scoring model that is straightforward and practical for predicting UTIs in patients with AIS. In general, our model exhibited a good overall performance and was comparatively more user-friendly and readily available for real clinical practice.

## Methods

### Study design and participants

This retrospective cohort study included consecutive patients with AIS who were admitted to the Tongde Hospital of Zhejiang Province, China, from January 1st, 2019 to December 31st, 2021. Only patients with AIS within 48 h of symptom onset, which was confirmed by computed tomography and/or magnetic resonance imaging, were included. Patients who developed an acute infection preceding stroke, were < 18 years of age, and individuals with incomplete or missing follow-up information that prevented subsequent analysis were excluded. Eligible patients admitted between January 1, 2019, and December 31, 2020, were included in the training cohort for the development of the model, and those who were admitted between January 1 and December 31, 2021, were included in the validation cohort.

The research followed the TRIPOD guidelines and was approved by the Ethics Committee of Tongde Hospital in Zhejiang Province (Acceptance number: 2023–139(K)). All procedures were performed in accordance with the principles of the Declaration of Helsinki. The requirement for informed consent was waived by the Ethics Committee of Tongde Hospital of Zhejiang Province because of its retrospective nature of the study.

### Data collection

Data were obtained from the electronic database of the hospital. Clinical and demographic data, including age, sex, seasonality, body mass index (BMI), smoking history, alcohol consumption, and medical history (including stroke, atrial fibrillation, coronary heart disease, hypertension, diabetes mellitus, hyperlipidemia, gout, and tumors), were collected. Initial data on admission were also collected, including consciousness level, NIHSS score, white blood cell (WBC) count, neutrophil count, lymphocyte count, and C-reactive protein (CRP). Additionally, post-stroke procedures such as thrombolysis, thrombectomy, urinary catheterization, and tracheotomy were documented. The NIHSS score^25^, which ranges from 0 (normal function) to 42 (death), was used to quantify stroke severity, with higher scores indicating more significant neurological deficit. The Neutrophil-to-lymphocyte ratio (NLR) was calculated by dividing the neutrophil count by the lymphocyte count.

### Primary outcomes

The primary outcome was the onset of UTIs within the first 14 days after AIS, which was defined as: positive midstream urine culture results with bacterial growth exceeding 10^5^ CFU/mL; presence of urinary tract symptoms such as urgency, frequency, dysuria, suprapubic tenderness, or fever (temperature > 38 ℃), indicating a possible UTI; and confirmation through positive findings in routine urinalysis. UTI diagnoses were adjudicated by physicians, and the detailed diagnostic criteria can be found in previous literatures^26,27^.

### Statistical analysis

Quantitative variables are presented as either the mean ± standard deviation (SD) or median (interquartile range [IQR]) and compared using the Student's t-test or Mann–Whitney U test. Qualitative data are described as percentages and analyzed using Pearson's chi-squared test or Fisher's exact test. Missing data was imputed by employing multiple imputations in the R package mice.

The significance of each variable in the training cohort was assessed through univariate logistic regression analysis. Backward stepwise regression was then performed using multivariate logistic analysis for significant variables (P < 0.05) identified in the univariate analysis. Risk variables with a P value < 0.05 were ultimately considered significant contributors to UTIs after stroke in the final model. A nomogram was created based on the multivariate analysis, incorporating the following four independent prognostic factors: age, sex, NIHSS score, and use of a urinary catheter. The discrimination of the model was assessed by calculating the concordance index (C statistic), which is equivalent to the area under the receiver operating characteristic (ROC) curve (AUC). Model calibration was visually presented in calibration plots and combined with the Hosmer–Lemeshow test. An insignificant result from the Hosmer–Lemeshow test also indicated good calibration (P > 0.05). The total score of each patient was finally calculated based on the nomogram. The optimal cut-off value for high UTIs risk was determined using the Youden index (Youden index = sensitivity + specificity − 1) derived from ROC analysis. Furthermore, the sensitivity, specificity, and accuracy were also calculated. Clinical utility was evaluated through the implementation of a decision curve analysis (DCA) curve. Statistical analyses were performed using R software (version 4.3.1). A two-tailed P-value of < 0.05 was considered statistically significant.

## Results

### Patient characteristics

**Figure 1:**
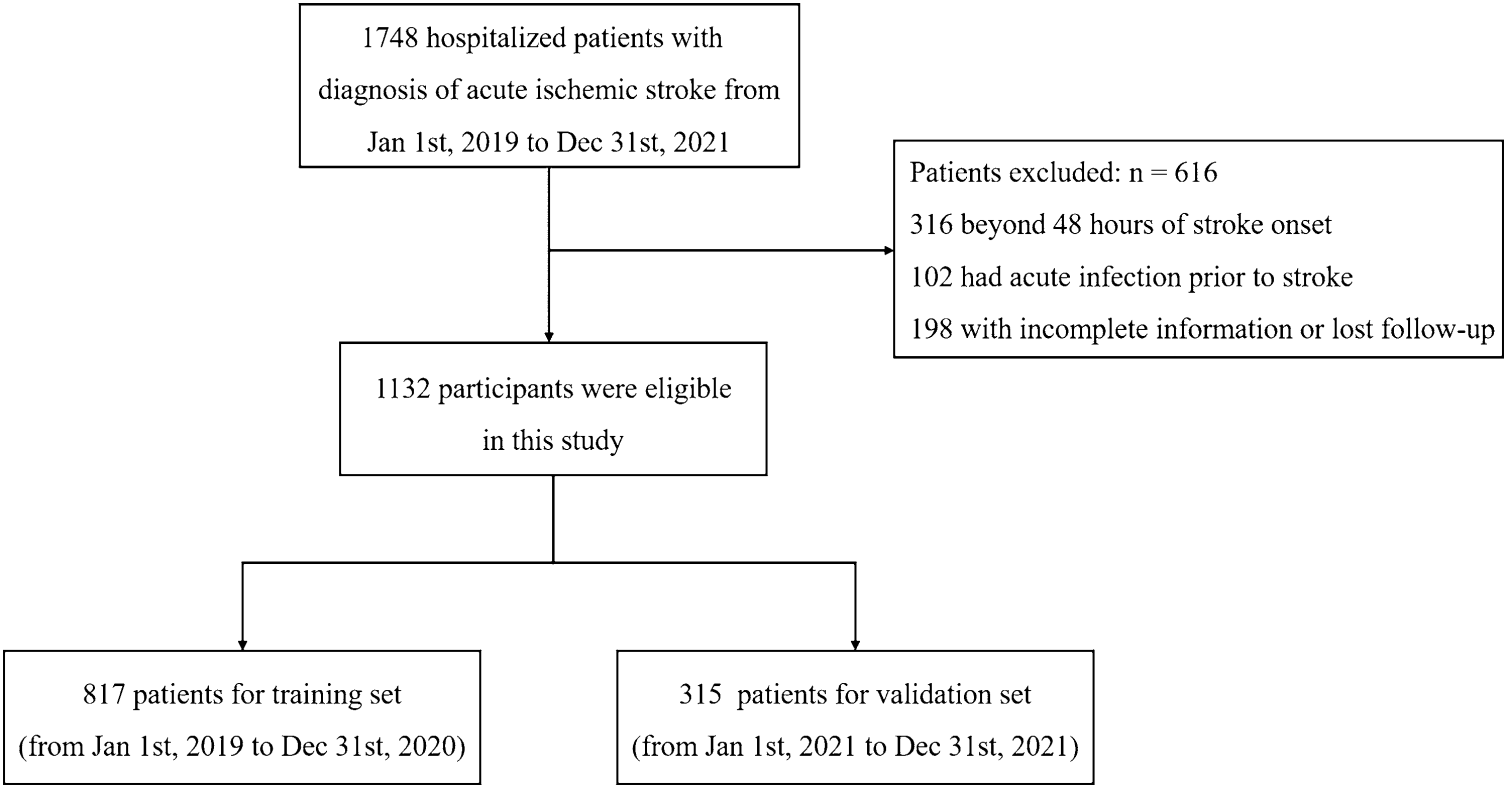
Study flow diagram.

In total, 1748 patients with AIS were admitted to our hospital. We excluded 316 individuals with AIS occurring beyond 48 h after onset, 102 who experienced an acute infection prior to stroke, and 198 individuals with incomplete information or who were lost to follow-up. Finally, we included a total of 1132 participants in the final analysis, with 817 participants used for model construction and 315 for external validation (Fig. 1).

**Figure 2:**
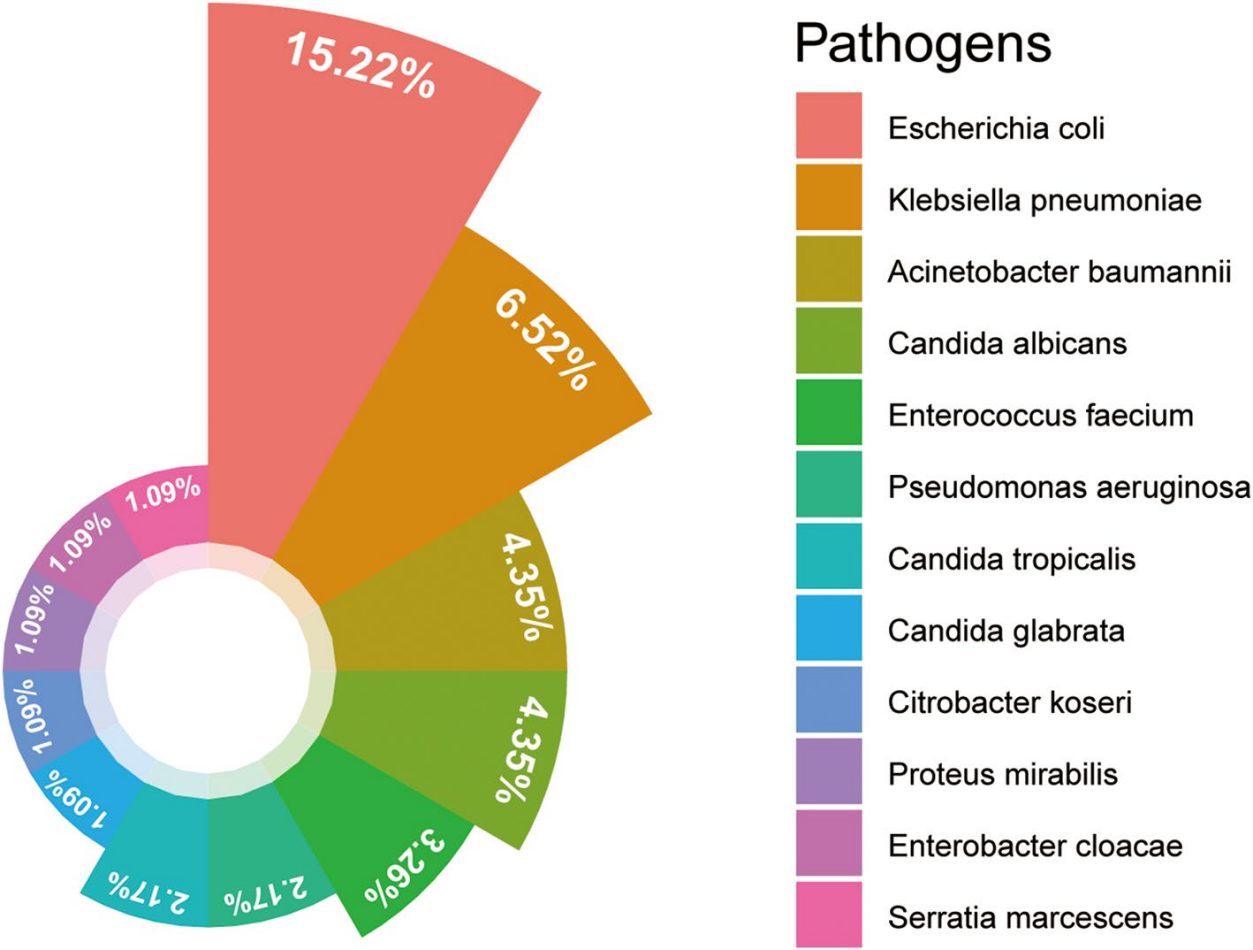
Nightingale rose chart displaying pathogens of urinary tract infections after acute ischemic stroke.

As demonstrated in Table 1, the baseline characteristics data were similar between the training and validation cohorts. The age of the patients was 68.99 ± 13.52 years, and 59.89% (678 out of 1132) were male. Over a mean follow-up period of 13.43 days, the overall incidence of UTIs was 8.13% (92/1132), with 7.59% (62/817) in the training cohort and 9.52% (30/315) in the validation cohort. The most prevalent pathogen responsible for post-AIS UTIs was *Escherichia coli* (15.22%, 14/92), followed by *Klebsiella pneumoniae* (6.52%, 6/92), *Acinetobacter baumannii* (4.35%, 4/92), and *Candida albicans* (4.35%, 4/92) (Fig. 2).

**Table 1:** Baseline characteristics of participants.

Variables	Total (n = 1132)	Training cohort (n = 817)	Validation cohort (n = 315)	P value^†^
Age, mean ± SD	68.99 ± 13.52	69.37 ± 13.49	68.01 ± 13.57	0.127
Sex, n (%)				0.052
Male	678 (59.89)	475 (58.14)	203 (64.44)	
Female	454 (40.11)	342 (41.86)	112 (35.56)	
Thrombolysis, n (%)	68 (6.01)	41 (5.02)	27 (8.57)	0.024
Thrombectomy, n (%)	19 (1.68)	15 (1.84)	4 (1.27)	0.506
Seasons, n (%)				0.016
Spring	277 (24.47)	190 (23.26)	87 (27.62)	
Summer	289 (25.53)	224 (27.42)	65 (20.63)	
Autumn	329 (29.06)	245 (29.99)	84 (26.67)	
Winter	237 (20.94)	158 (19.34)	79 (25.08)	
BMI, Mean ± SD^‡^^,§^	23.82 ± 3.63	23.74 ± 3.74	24.02 ± 3.33	0.248
Smoker, n (%)	362 (31.98)	262 (32.07)	100 (31.75)	0.917
Drinker, n (%)	328 (28.98)	249 (30.48)	79 (25.08)	0.073
Stroke, n (%)	226 (19.96)	155 (18.97)	71 (22.54)	0.178
Atrial fibrillation, n (%)	168 (14.84)	124 (15.18)	44 (13.97)	0.608
Coronary heart disease, n (%)	165 (14.58)	124 (15.18)	41 (13.02)	0.356
Hypertension, n (%)	871 (76.94)	631 (77.23)	240 (76.19)	0.709
Diabetes, n (%)	365 (32.24)	259 (31.7)	106 (33.65)	0.529
Hyperlipidemia, n (%)	148 (13.07)	103 (12.61)	45 (14.29)	0.453
Gout, n (%)	45 (3.98)	27 (3.30)	18 (5.71)	0.063
Tumour, n (%)	70 (6.18)	50 (6.12)	20 (6.35)	0.886
NIHSS score, n (%)				0.062
< 5	836 (73.85)	591 (72.34)	245 (77.78)	
≥ 5	296 (26.15)	226 (27.66)	70 (22.22)	
Consciousness, n (%)	136 (12.01)	107 (13.1)	29 (9.21)	0.071
Urinary catheter, n (%)	149 (13.16)	110 (13.46)	39 (12.38)	0.629
Tracheotomy, n (%)	38 (3.36)	32 (3.92)	6 (1.90)	0.092
UTI, n (%)	92 (8.13)	62 (7.59)	30 (9.52)	0.286
WBC, Mean ± SD^‡^	7.24 ± 2.54	7.20 ± 2.55	7.32 ± 2.52	0.479
NLR, Median (IQR)^‡^^,^^¶^	2.80 (1.95, 4.50)	2.80 (1.96, 4.50)	2.84 (1.90, 4.40)	0.918
CRP, Median (IQR)^‡^	1.99 (0.80, 5.29)	2.10 (0.80, 5.80)	1.70 (0.76, 4.20)	0.036

*SD* standard deviation, *IQR* interquartile range, *BMI* body mass index, *NIHSS* the National Institutes of Health Stroke Scale, *UTI* Urinary tract infection, *WBC* White blood cell count, *NLR* Neutrophil to lymphocyte ratio, *CRP* C-reactive protein.

^†^P value was based on Student’s t-test, Mann–Whitney U test, χ2 tests, or Fisher’s exact, as appropriate.

^‡^Missing data: 38 for BMI, 15 for WBC, 15 for NLR, and 29 for CRP.

^§^BMI was calculated as weight in kilograms divided by height in meters squared.

^¶^NLR was calculated as the neutrophil count divided by the lymphocyte count.

### Model derivation

We included a total of 23 established risk factors as candidate variables, all of which were used for univariate logistic regression analysis (Table 2). The backward stepwise selection using multivariate logistic analysis identified four variables that showed the strongest association with the risk of UTIs after AIS: advanced age (OR, 1.05; 95% CI 1.02–1.07; P < 0.001), female gender (OR, 2.10; 95% CI 1.17–3.77; P = 0.013), NIHSS score ≥ 5 (OR, 2.28; 95% CI 1.18–4.41; P = 0.015), and indwelling urinary catheters (OR, 3.43; 95% CI 1.75–6.74; P < 0.001) (Table 2).

**Table 2:** Univariate and multivariate logistic regression analysis of UTI presence based on data in the training cohort.

Variable	Univariable		Multivariable	
	OR (95% CI)	P value	OR (95% CI)	P value
Age, y	1.07 (1.04 ~ 1.10)	< 0.001	1.05 (1.02 ~ 1.07)	< 0.001
Sex, female vs. male	2.94 (1.71 ~ 5.08)	< 0.001	2.10 (1.17 ~ 3.77)	0.013
Thrombolysis, yes vs. no	1.75 (0.66 ~ 4.64)	0.259		
Thrombectomy, yes vs. no	1.90 (0.42 ~ 8.63)	0.404		
Seasons, summer vs. spring	0.97 (0.46 ~ 2.04)	0.930		
Seasons, autumn vs. spring	0.88 (0.42 ~ 1.85)	0.732		
Seasons, winter vs. spring	1.42 (0.67 ~ 3.00)	0.363		
BMI	0.92 (0.85 ~ 0.99)	0.034		
Smoker, yes vs. no	1.01 (0.58 ~ 1.76)	0.973		
Drinker, yes vs. no	0.65 (0.35 ~ 1.19)	0.163		
Stroke, yes vs. no	1.54 (0.85 ~ 2.80)	0.156		
Atrial fibrillation, yes vs. no	2.29 (1.26 ~ 4.14)	0.006		
Coronary heart disease, yes vs. no	1.54 (0.81 ~ 2.93)	0.189		
Hypertension, yes vs. no	1.25 (0.65 ~ 2.40)	0.506		
Diabetes, yes vs. no	0.87 (0.49 ~ 1.54)	0.639		
Hyperlipidemia, yes vs. no	0.46 (0.16 ~ 1.29)	0.138		
Gout, yes vs. no	2.19 (0.73 ~ 6.56)	0.159		
Tumour, yes vs. no	1.73 (0.71 ~ 4.24)	0.230		
NIHSS score, ≥ 5 vs. < 5	5.15 (3.00 ~ 8.85)	< 0.001	2.28 (1.18 ~ 4.41)	0.015
Consciousness, yes vs. no	3.98 (2.25 ~ 7.06)	< 0.001		
Urinary catheter, yes vs. no	7.31 (4.22 ~ 12.67)	< 0.001	3.43 (1.75 ~ 6.74)	< 0.001
Tracheotomy, yes vs. no	3.00(1.19 ~ 7.60)	0.020		
WBC, × 10^9^/L	1.12 (1.03 ~ 1.22)	0.011		
NLR	1.10 (1.04 ~ 1.16)	< 0.001		
CRP, mg/L	1.01 (1.00 ~ 1.02)	0.006		

*BMI* body mass index, *NIHSS* the National Institutes of Health Stroke Scale, *UTI* Urinary tract infection, *WBC* White blood cell count, *NLR* Neutrophil to lymphocyte ratio, *CRP* C-reactive protein, *OR* odds ratio, *CI* confidence interval.

**Figure 3:**
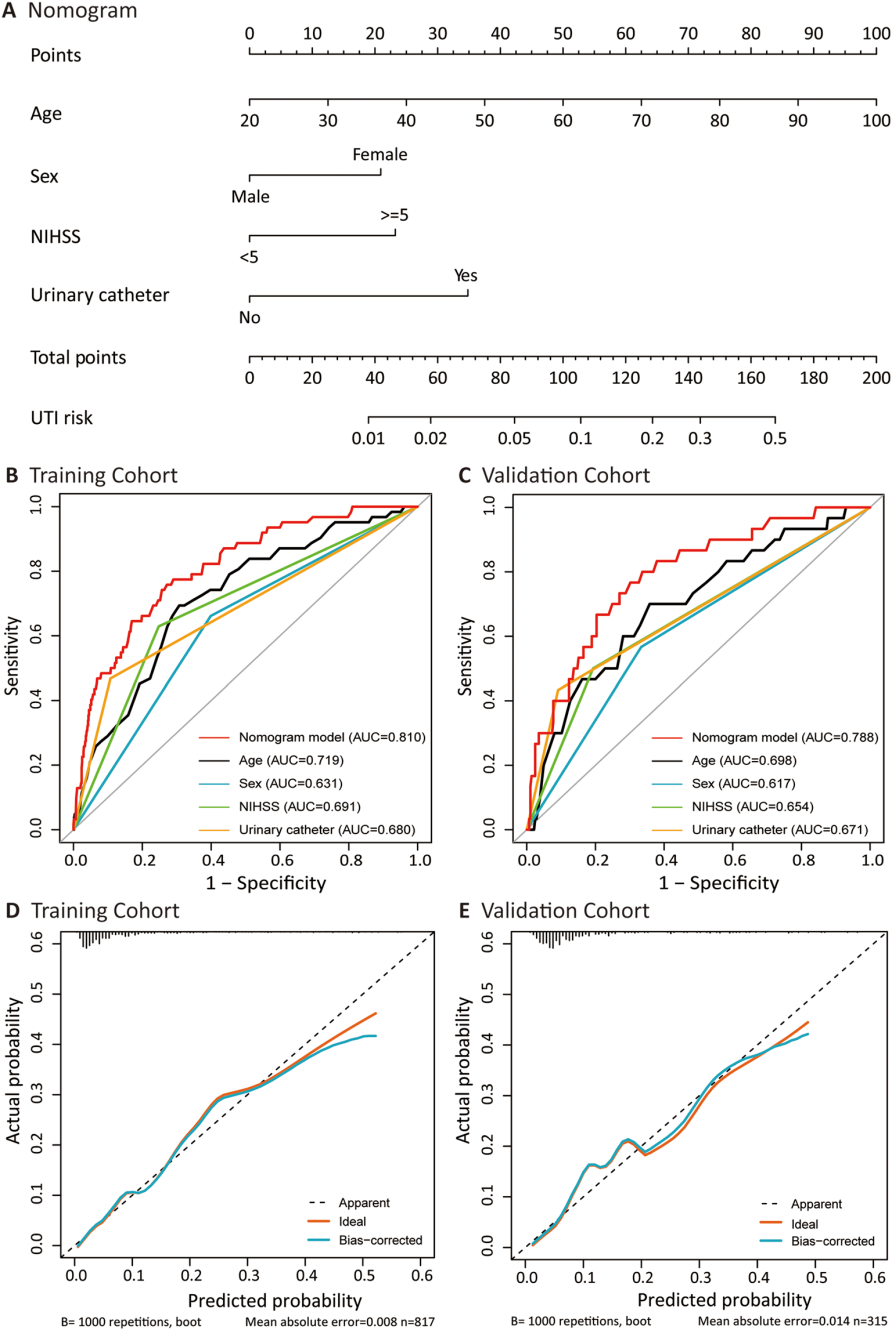
(**A**) Nomogram for predicting urinary tract infections in patients after acute ischemia stroke. (**B**) The ROC curves for training cohort. (**C**) The ROC curves for validation cohort. (**D**) The calibration plot for training cohort. (**E**) The calibration plot for validation cohort.

Subsequently, a nomogram was constructed (Fig. 3A) and scored based on these four factors (Table 3). The higher the total score, the higher the risk of UTIs. The risk of UTIs was considered high when the nomogram predicted a probability of ≥ 6.98%, corresponding to a total score of 95. The correlation between the prediction probability of post-AIS UTIs and their corresponding total score is illustrated in Supplementary Table S1. Our nomogram-based score is referred to as the "SUNA" score, which is an acronym derived from the initial letters of four key risk factors: sex, utilization of urinary catheter, NIHSS score, and age.

**Table 3:** SUNA score for predicting the risk of urinary tract infections after acute ischemic stroke.

Variables	Points
Sex (Female)	21
Urinary catheter utilization	35
NIHSS score ≥ 5	23
Age	1.25*Age-25

High UTI risk was considered when the nomogram predicted probability was ≥ 6.98% (corresponding to a total score of 95).

For example, an 80-year-old male patient, with an NIHSS score of 8 had an indwelling urinary catheter during the follow-up period.

Total score = 35 (indwelled urinary catheter) + 23 (NIHSS ≥ 5) + 75 (1.25 * 80 (age) – 25) = 133 points.

Therefore, this patient had a total score of 133, which is ≥ 95, and belonged to the high-risk group for UTIs.

### Model validation

The model achieved a C index (AUC) of 0.810 (95% CI, 0.756–0.865) in the training cohort and 0.788 (95% CI 0.704–0.873) in the validation cohort, demonstrating good discriminatory ability. Meanwhile, both ROC curves demonstrated that the nomogram model established in this study had a superior AUC than any individual risk factor (Fig. 3B and C). The Hosmer–Lemeshow fit was acceptable, with a P-value of 0.811 in the training set and 0.192 in the validation set. Furthermore, the calibration plots obtained via 1000 bootstrap resamples exhibited satisfactory calibration of the model (Fig. 3D and E).

### Evaluation of the clinical applicability of the model

**Figure 4:**
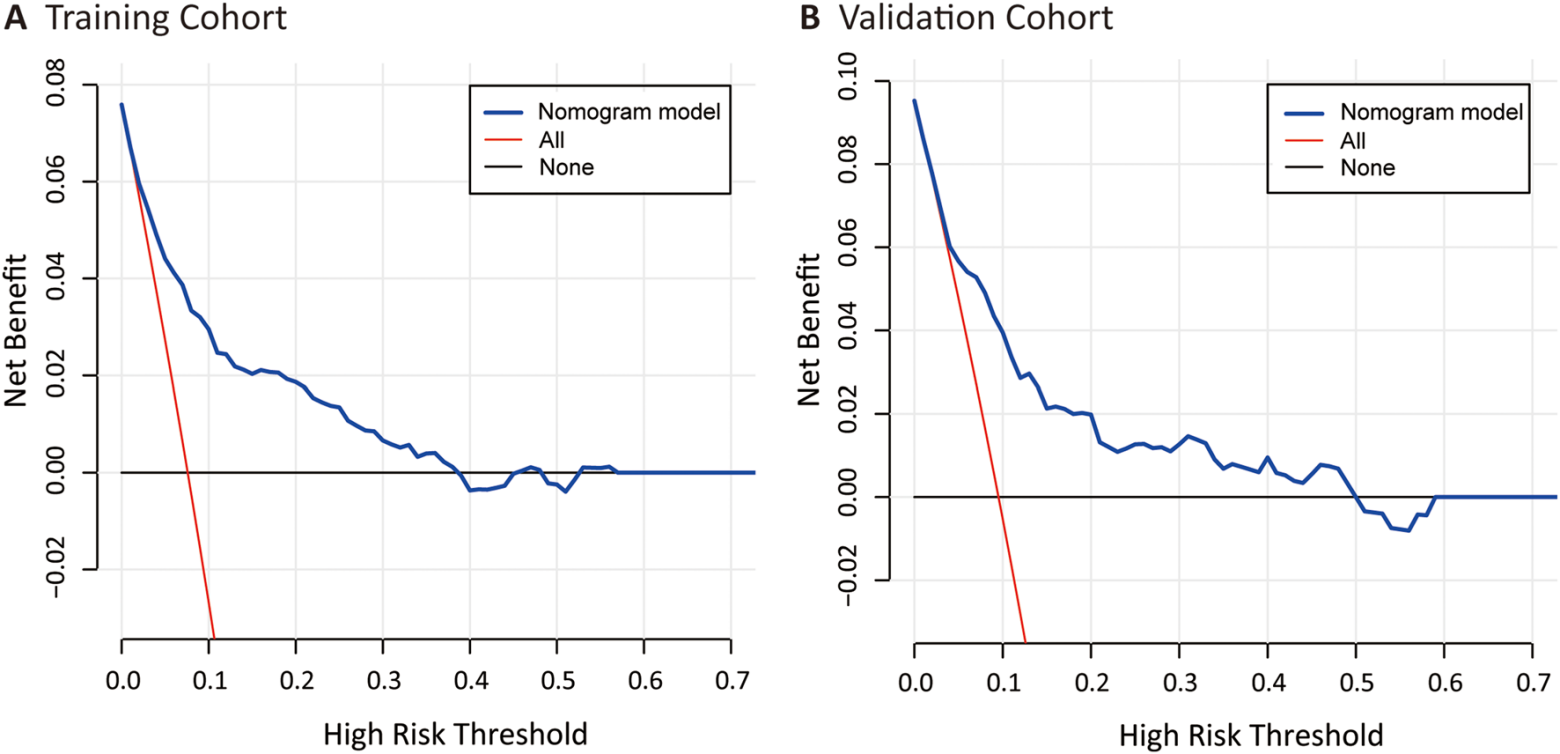
(**A**) Decision curve analysis for training cohort. (**B**) Decision curve analysis for validation cohort.

The decision curves show the net benefits gained from applying our model across a range of relevant risk thresholds (Fig. 4). Specifically, the DCA in the validation set demonstrated that, if the threshold probability was between 0.05 and 0.5, utilizing the developed model to predict UTIs risk in patients with AIS was more beneficial than focusing on either all or no patients. Given that the incidence of post-stroke UTI is approximately 2–27%, which falls within the range of threshold probabilities, this model is considered to have good clinical utility.

The optimal cutoff value for the total nomogram score was 95. At the optimal cutoff value, the sensitivity, specificity, and accuracy were 75.8%, 72.8%, and 73.1%, respectively in the training cohort, and 76.7%, 69.8%, and 70.5%, respectively in the validation cohort (Table 4).

**Table 4:** Accuracy of the prediction score of the Nomogram.

Variable	Value (95% CI)	
	Training cohort	Validation cohort
Area under ROC curve, concordance index	0.810 (0.756–0.865)	0.788 (0.704–0.873)
Nomogram predicted probability	6.98%	6.98%
Cutoff score	95	95
Sensitivity (95% CI), %	75.8 (65.1–86.5)	76.7 (61.5–91.8)
Specificity (95% CI), %	72.8 (69.7–76.0)	69.8 (64.5–75.2)
Accuracy (95% CI), %	73.1 (73.0–73.1)	70.5 (70.3–70.6)

*ROC* receiver operating characteristic, *CI* confidence interval.

## Discussion

Post-stroke UTI is one of the most prevalent complications of stroke; however, easily applicable and reliable models for the early prediction of UTIs risk in patients with AIS are scarce. In this observational real-world cohort study, we conducted a comprehensive analysis of 1132 consecutive patients and successfully developed and validated a novel, user-friendly nomogram-based scoring model that incorporates four readily accessible clinical variables: age, sex, NIHSS score, and use of a urinary catheter. Finally, the model exhibited satisfactory discrimination and accuracy, and possessed considerable clinical utility in predicting UTIs among patients with AIS.

In the current study, UTIs were observed in 8.13% of patients with AIS, which is consistent with previous reports that have documented incidence rates ranging from 2 to 27%^8–10^. The variability in incidence rates is probably attributable to variations in follow-up duration, sample sizes, stroke subtype, populations studied, and the definition of UTIs^8,28^. Among previous studies, the follow-up period has been reported to range from 48 hours^29,30^ to 30 months^31^ post-stroke, with the majority of studies having a sample size of less than 1000. Meanwhile, the stroke type comprised ischemic, hemorrhagic, or mixed stroke (both ischemic and hemorrhagic), and a previous study demonstrated an elevated risk of UTIs in individuals with a mixed stroke type^24^. In general, patients with longer follow-up durations^31^, those admitted to neurological intensive care units^32^, and immobile patients in rehabilitation settings^33^ demonstrate higher frequencies of post-stroke infections. Although the reported frequencies of UTIs vary widely, the estimated incidence of UTIs in patients with acute phase stroke is still commonly cited at 10%^13,34^. Our study found an incidence rate of 8.13%, which was almost similar to this incidence rate. The following factors may explain why our incidence rate was slightly lower than 10%: the follow-up duration, stroke type being AIS rather than a mix of stroke types, and the study population comprising all patients instead of exclusively focusing on individuals receiving intensive care or immobile patients with stroke.

Our study also indicates that clinical factors, such as advanced age, female gender, elevated NIHSS score, and utilization of urinary catheters, are significantly associated with post-stroke UTIs. These results were consistent with those of most previous studies^15–17^. Decreased immune function and increased comorbidities of the urinary system may contribute to a higher frequency of UTIs in older patients with stroke^7^. Females are more susceptible to infection, primarily because of anatomical differences in the urethra between men and women, where a shorter distance between the urethral and anal openings may increase vulnerability^35^. According to our current analysis, an NIHSS score of ≥ 5 upon initial admission was identified as an independent predictor, which is consistent with previous studies that have reported a similar association^36^. This association may also be attributed to stroke-induced immune suppression, which predisposes patients to bacterial infections^7^. Consistent with our findings, several studies have also observed significant disparities in urinary catheter utilization between patients with or without UTIs^12,14,17^. Therefore, avoiding unnecessary catheterization is likely to effectively prevent post-stroke UTIs^37^. In addition, significant differences were observed in several other variables during the univariate analysis, including WBC count, NLR, CRP, BMI, history of atrial fibrillation, consciousness level, and tracheotomy. However, these variables were excluded during the stepwise regression analyses, enabling us to identify the most significant variables for predicting post-stroke UTIs.

To date, limited predictive models for post-AIS UTIs have been established, and none of them has been developed as a scoring system. In a study conducted by Li et al.^22^, a logistic regression model was constructed to predict UTIs in patients after AIS, incorporating four influencing factors (sex, NIHSS score, interleukin-6, and hemoglobin). Sex and NIHSS score were consistent risk factors between their model and ours. However, the logistic model had a limited sample size of only 186 participants and lacked calibration and external validation. Therefore, the obtained conclusions may not be rigorous enough. Another study^24^ developed six machine learning models and an ensemble learning model to assess the risk of post-stroke UTIs using a large sample size. However, the study was conducted exclusively on immobile patients with stroke, rather than on all stroke patients. So, the study population was different from ours. One additional recent study^23^ used several machine learning models to predict UTIs after stroke. Whereas, the study population exclusively comprised patients with hemorrhagic stroke identified from a specialized neuro-intensive care unit, which also differed from the cohort in our study. Additionally, the generalizability of this study may be limited due to its relatively small sample size (n = 110) and the necessity for specific computer software and sophisticated algorithms. Moreover, none of these three studies evaluated the clinical utility of these models. Overall, compared to existing models, our study focuses on all patients with AIS and boasts a relatively large sample size. Meanwhile, our nomogram-based scoring system contains only four concise risk factors, making it more user-friendly and readily applicable in clinical practice than the existing models. A simplified and easy-to-remember post-AIS UTIs risk score enhances its suitability for bedside diagnosis, enabling clinicians to promptly identify high-risk patients without the need for laboratory testing, ancillary assessments, additional software, or complex algorithms. Besides, conducting more extensive model validation and further evaluating the clinical utility will enhance the reliability of our study.

The model constructed in this study can assist in therapeutic decision making. A nomogram score above the cutoff value of 95 identifies candidates who are at high risk of developing post-stroke UTIs and require a prompt response to potential UTIs. Although no data are available regarding the recommended prophylactic antibiotic therapy^7,38^, further investigation is necessary to determine the appropriate interventions for this specific patient population, including the implementation of comprehensive care within a stroke unit^7,39,40^. In contrast, a nomogram score < 95 may not require routine urinalysis, as our model demonstrates a relatively low risk of UTIs, and regular screening for UTIs may offer limited benefits. Furthermore, the model supports the notion that early removal of urinary catheters, when possible, constitutes an effective strategy for reducing the incidence of UTIs after stroke. The simplicity and user-friendliness of our scoring system make it highly suitable for bedside diagnosis, thus positioning it as an ideal fit for all medical institutions, particularly primary care facilities with limited resources. Meanwhile, the model we have developed is particularly well-suited for predicting the risk of UTIs in patients with AIS, and it demonstrates superior performance in predicting UTIs occurring within 14 days. In summary, the model enables healthcare teams to intervene proactively and customize treatments for individual patients, ultimately enhancing patient care, reducing complications, and mitigating healthcare costs.

The major strength of this study is that it constructed the first novel, user-friendly nomogram-based scoring model to explicitly predict UTI risk within 14 days in patients with AIS in general wards. Both training and validation sets were adequately powered to demonstrate good discrimination and calibration. Additionally, all the risk factors incorporated into the model can be assessed upon admission without relying on laboratory indicators or imaging examinations, making it easier to use. Furthermore, this study relies on data from a tertiary hospital cohort with a well-established electronic medical record system to ensure data reliability. However, this study has some potential limitations. First, this was a single-center study in China; therefore, data from multiple centers in wider geographic regions are required to validate the model’s performance. Second, the retrospective cohort design may have introduced inherent selection bias, highlighting the need for prospective studies to validate our findings. Third, these data are more applicable for predicting UTI risk within 14 days in patients with AIS. Therefore, caution should be exercised when generalizing the results to other stroke types or follow-up periods. Fourth, the training cohort and validation cohort exhibit statistically significant differences in Thrombolysis, Seasons, and CRP, which may impact the balance between the two groups and subsequently affect the generalizability and predictive accuracy of the nomogram model. Although these variables are not incorporated into the model, the inclusion of other validation cohorts would enhance the widespread applicability of the model.

In conclusion, we developed a novel and user-friendly nomogram-based scoring model for predicting the risk of UTIs in patients with AIS using simple and readily available variables. The model showed favorable performance and clinical utility. These findings provide valuable information for timely intervention in patients with AIS, aiming to reduce the occurrence of UTIs and subsequently improve the outcomes of AIS.

### Supplementary Information

Supplementary Table S1.

## Data Availability

The data used in this study may be available from the corresponding author upon reasonable request.

## References

[1] World Health Organization. The top 10 causes of death. https://www.who.int/news-room/fact-sheets/detail/the-top-10-causes-of-death (2020).

[2] Kleindorfer DO (2021). 2021 guideline for the prevention of stroke in patients with stroke and transient ischemic attack: A guideline from the American Heart Association/American Stroke Association. Stroke.

[3] Campbell BCV, Khatri P (2020). Stroke. Lancet.

[4] Virani SS (2020). Heart disease and stroke statistics-2020 update: A report from the American Heart Association. Circulation.

[5] Ingeman A, Andersen G, Hundborg HH, Svendsen ML, Johnsen SP (2011). In-hospital medical complications, length of stay, and mortality among stroke unit patients. Stroke.

[6] Kumar S, Selim MH, Caplan LR (2010). Medical complications after stroke. Lancet Neurol..

[7] Westendorp WF, Dames C, Nederkoorn PJ, Meisel A (2022). Immunodepression, infections, and functional outcome in ischemic stroke. Stroke.

[8] Emsley HCA, Hopkins SJ (2008). Acute ischaemic stroke and infection: Recent and emerging concepts. Lancet Neurol..

[9] Ahmed R (2023). Age- and sex-specific trends in medical complications after acute ischemic stroke in the United States. Neurology.

[10] Wang PL (2012). Effect of in-hospital medical complications on case fatality post-acute ischemic stroke: data from the China National Stroke Registry. Chin. Med. J..

[11] Poisson SN, Johnston SC, Josephson SA (2010). Urinary tract infections complicating stroke: Mechanisms, consequences, and possible solutions. Stroke.

[12] Stott DJ, Falconer A, Miller H, Tilston JC, Langhorne P (2009). Urinary tract infection after stroke. Qjm-Int. J. Med..

[13] Smith C, Almallouhi E, Feng W (2019). Urinary tract infection after stroke: A narrative review. J. Neurol. Sci..

[14] Jitpratoom P, Boonyasiri A (2023). Determinants of urinary tract infection in hospitalized patients with acute ischemic stroke. BMC Neurol..

[15] Wästfelt M, Cao Y, Ström JO (2018). Predictors of post-stroke fever and infections: A systematic review and meta-analysis. BMC Neurol..

[16] Mu J (2020). A retrospective study on risk factors for urinary tract infection in patients with intracranial cerebral hemorrhage. Biomed Res. Int..

[17] Zhu C (2020). Prevalence, incidence, and risk factors of urinary tract infection among immobile inpatients in China: A prospective, multi-centre study. J. Hosp. Infect..

[18] Chen Y (2022). Prediction model for urinary tract infection in pediatric urological surgery patients. Front. Public Health.

[19] Li Y (2023). Development and validation of a user-friendly risk nomogram for the prediction of catheter-associated urinary tract infection in neuro-intensive care patients. Intens. Crit. Care Nur..

[20] Shaikh N (2018). Development and validation of a calculator for estimating the probability of urinary tract infection in young febrile children. JAMA Pediatri..

[21] Taylor RA, Moore CL, Cheung KH, Brandt C (2018). Predicting urinary tract infections in the emergency department with machine learning. Plos One.

[22] Li YM, Xu JH, Zhao YX (2020). Predictors of urinary tract infection in acute stroke patients: A cohort study. Medicine.

[23] Zhao Y (2023). Prediction of upcoming urinary tract infection after intracerebral hemorrhage: A machine learning approach based on statistics collected at multiple time points. Front. Neurol..

[24] Zhu C (2022). Prediction of post-stroke urinary tract infection risk in immobile patients using machine learning: An observational cohort study. J. Hosp. Infect..

[25] Lyden P (2005). NIHSS training and certification using a new digital video disk is reliable. Stroke.

[26] Groen J (2016). Summary of European Association of Urology (EAU) guidelines on neuro-urology. Eur. Urol..

[27] Horan TC, Andrus M, Dudeck MA (2008). CDC/NHSN surveillance definition of health care-associated infection and criteria for specific types of infections in the acute care setting. Am. J. Infect. Control.

[28] Yan T (2018). Prevalence and predictive factors of urinary tract infection among patients with stroke: A meta-analysis. Am. J. Infect. Control..

[29] Grau AJ (1999). Fever and infection early after ischemic stroke. J. Neurol. Sci..

[30] Wong AA (2007). The time course and determinants of temperature within the first 48 h after ischaemic stroke. Cerebrovasc. Dis..

[31] Langhorne P (2000). Medical complications after stroke: A multicenter study. Stroke.

[32] Yilmaz GR, Cevik MA, Erdinc FS, Ucler S, Tulek N (2007). The risk factors for infections acquired by cerebral hemorrhage and cerebral infarct patients in a neurology intensive care unit in Turkey. JPN. J. Infect. Dis..

[33] Ersoz M, Ulusoy H, Oktar MA, Akyuz M (2007). Urinary tract infection and bacteriurua in stroke patients: Frequencies, pathogen microorganisms, and risk factors. Am. J. Phys. Med. Rehab..

[34] Westendorp WF, Nederkoorn PJ, Vermeij JD, Dijkgraaf MG, van de Beek D (2011). Post-stroke infection: A systematic review and meta-analysis. BMC Neurol..

[35] Flores-Mireles AL, Walker JN, Caparon M, Hultgren SJ (2015). Urinary tract infections: Epidemiology, mechanisms of infection and treatment options. Nat. Rev. Microbiol..

[36] Chen SC (2018). Portable bladder ultrasound reduces incidence of urinary tract infection and shortens hospital length of stay in patients with acute ischemic stroke. J. Cardiovasc. Nurs..

[37] Kunin CM (2006). Urinary-catheter-associated infections in the elderly. Int. J. Antimicrob. Ag..

[38] Westendorp WF (2015). The Preventive Antibiotics in Stroke Study (PASS): A pragmatic randomised open-label masked endpoint clinical trial. Lancet.

[39] Stroke Unit Trialists' Collaboration (2013). Organised inpatient (stroke unit) care for stroke. Cochrane. DB. Syst. Rev..

[40] Govan L, Langhorne P, Weir CJ (2007). Does the prevention of complications explain the survival benefit of organized inpatient (stroke unit) care?: Further analysis of a systematic review. Stroke.

