# Supplementary material for: A novel score for early prediction of urinary tract infection risk in patients with acute ischemic stroke: a nomogram-based retrospective cohort study
Source: Sci Rep. 2024 May 10;14:10707. doi: 10.1038/s41598-024-61623-0 (PMC11087532; doi:10.1038/s41598-024-61623-0)
Supplement: Supplementary file 1 — Supplementary Table S1. [file 41598_2024_61623_MOESM1_ESM.pdf]

A novel score for early prediction of urinary tract infection risk in patients with acute ischemic stroke: a nomogram-based retrospective cohort study

Qinqin Zhao[1], Pinpin Feng[1], Jun Zhu[1], Yunling Wang[2], Xiaojuan Zhou[1], Zhongni Xia[1], Danqing Wang[3], Yueyue He[3], Pei Wang[1*], Xiang Li[4*]

Supplementary Table S1 The prediction probability of UTIs and their corresponding total score.

Prediction probability	Total score	Prediction probability	Total score
0.01	38	0.32	146
0.02	58	0.33	148
0.03	70	0.34	149
0.04	78	0.35	150
0.05	85	0.36	151
0.06	90	0.37	153
0.07	95	0.38	154
0.08	99	0.39	155
0.09	102	0.40	156
0.10	106	0.41	157
0.11	109	0.42	159
0.12	111	0.43	160
0.13	114	0.44	161
0.14	116	0.45	162
0.15	119	0.46	163
0.16	121	0.47	164
0.17	123	0.48	165
0.18	125	0.49	167
0.19	127	0.50	168
0.20	129	0.51	169
0.21	130	0.52	170
0.22	132	0.53	171
0.23	134	0.54	172
0.24	135	0.55	173
0.25	137	0.56	175
0.26	138	0.57	176
0.27	140	0.58	177
0.28	141	0.59	178
0.29	142	0.60	179
0.30	144	0.61	180
0.31	145	0.62	182
